# The Effect of Sleep Duration on Hypertension Risk in an Adult Asian Population: A Systematic Review and Meta-Analysis

**DOI:** 10.7759/cureus.61508

**Published:** 2024-06-01

**Authors:** Yusuf Aji S Nurrobi, Kevin Winston, Ivan Damara, Andi L Rahman, Moh F Falakhi, Meutia P Aristya, Ahmad F Toaha, Iva N Larasaty

**Affiliations:** 1 Cardiology, Pertamina Hospital, Balikpapan, IDN; 2 Cardiology, Faculty of Medicine, Universitas Airlangga, Surabaya, IDN; 3 Hospital Medicine, Bhakti Medicare Hospital, Cicurug, IDN; 4 Research, Oxford University Clinical Research Unit Indonesia, Jakarta, IDN; 5 General Medicine, Hasri Ainun Habibie Regional Hospital, Parepare, IDN; 6 General Medicine, Muhammadiyah Gresik Hospital, Gresik, IDN; 7 General Medicine, Metropolitan Medical Centre Hospital, Jakarta, IDN; 8 General Medicine, Labuang Baji Hospital, Makassar, IDN; 9 General Medicine, Halu Oleo University, Kendari, IDN

**Keywords:** meta-analysis, asian, adult, hypertension, sleep duration

## Abstract

Sleep duration has been proposed as a potential and important modifiable risk factor, yet its precise relationship with hypertension among Asian adults remains unclear. This meta-analysis aims to elucidate the impact of short sleep duration on hypertension risk within the adult Asian population. A systematic search of databases, including PubMed, Scopus, and ScienceDirect, was conducted to identify relevant studies published up to January 4, 2024. Eligible studies comprised observational cohort studies and cross-sectional studies that compared short sleep duration to normal sleep duration in relation to hypertension risk among Asian adults. The definitions for short and normal sleep durations were derived from the respective studies. The random effects model was utilized to pool effect estimates, and all statistical analyses were conducted using Review Manager 5.4 software (RevMan) (Cochrane Collaboration, Oxford, UK). Results from a systematic search obtained seven studies assessing sleep duration and hypertension risk in Asian populations. Based on a meta-analysis of six studies, short sleep duration is associated with a higher hypertension risk when compared to normal sleep duration (OR: 1.36; 95% CI: 1.13-1.64; p: 0.0010; I^2^: 75%). Subgroup analysis based on sex showed that the association is evident across males (OR: 1.12; 95% CI: 1.01-1.25; p: 0.03; I^2^: 64%) and females (OR: 1.22; 95% CI: 1.10-1.35; p: 0.0003; I^2^: 82%). In conclusion, based on the analyzed studies, short sleep duration is associated with a higher mild risk of hypertension, irrespective of sex. Thus, short sleep duration can be a modifiable risk factor that can be prevented to reduce the risk of hypertension. By incorporating sleep hygiene practices and promoting healthy sleep habits, significant improvement in cardiovascular health can be made, especially in hypertension risk at a population level. Further studies on the effect of sleep duration in different age populations should be conducted to confirm the impact of short sleep duration.

## Introduction and background

Hypertension presents a significant global health challenge, affecting over 30% of the adult population worldwide [1]. This condition not only impacts cardiovascular health but also strains healthcare systems, highlighting the importance of understanding its various determinants. Among these factors, sleep duration has garnered attention for its potential role in influencing hypertension risk, reflecting an evolving understanding of its significance in cardiovascular health [2].

Hypertension is often defined as systolic blood pressure ≥140 mmHg or diastolic blood pressure ≥90 mmHg. However, it should be noted that the criteria for diagnosing hypertension vary among different guidelines. The 2017 American College of Cardiology/American Heart Association (ACC/AHA) guidelines define hypertension as systolic blood pressure (SBP) ≥130 mmHg, diastolic blood pressure (DBP) ≥80 mmHg, or current use of antihypertensive medications [3]. In contrast, the 2023 European Society of Cardiology/European Society of Hypertension (ESC/ESH) guidelines maintain the traditional threshold of ≥140/90 mmHg for diagnosing hypertension [4].

The impact of insufficient sleep, particularly its association with hypertension, has drawn considerable interest on a global scale. Meta-analyses have revealed compelling evidence indicating that individuals with shorter sleep durations - those obtaining less than seven hours per night - face a 1.2-fold higher risk of developing hypertension compared to their well-rested counterparts [5]. Furthermore, each additional hour of sleep appears to confer a modest but noticeable reduction (approximately 0.32%) in hypertension risk [5]. Importantly, this heightened risk persists even after accounting for factors such as obesity and diabetes, underscoring the independent influence of inadequate sleep on hypertension susceptibility [6]. 

While numerous studies have explored the link between inadequate sleep and hypertension, there remains a notable gap in research focusing specifically on the Asian population [7-10]. Existing meta-analyses predominantly include diverse global cohorts, leaving a gap in understanding of how sleep duration impacts hypertension risk within Asian demographics [11].

Interestingly, a prevalent trend observed across various Asian regions, particularly in East Asian countries like Singapore, Hong Kong, and South Korea, is the presence of a tendency toward shorter sleep durations. This trend is influenced by cultural factors and heightened demands in professional and educational spheres, especially among adolescents [12]. Research also indicates that Asians have a higher likelihood of reporting short sleep duration compared to Whites, with a prevalence of 33% among Asians [13]. These studies collectively highlight the importance of addressing short sleep duration among Asians to improve overall health outcomes. Therefore, this systematic review and meta-analysis aim to address this gap by comprehensively evaluating and synthesizing available evidence, with a specific emphasis on elucidating the relationship between insufficient sleep duration and hypertension risk among Asian adults.

## Review

Methods

Eligibility Criteria

This study aimed to investigate the association between sleep duration and hypertension risk in Asian adult populations through observational cohort and cross-sectional studies. The formulated research question for this review is "Does short sleep duration in the Asian population cause hypertension?"

Inclusion criteria encompassed studies with clear definitions of short and normal sleep durations, reporting hypertension risk data with odds ratios (ORs) and their corresponding 95% confidence intervals (CIs), and publication in English. Exclusion criteria included studies not adhering to the observational cohort or cross-sectional design, studies where hypertension was present at baseline, research involving non-Asian populations, and studies not published in English.

Search Strategy

We conducted comprehensive searches across multiple databases including PubMed, Scopus, and EBSCOhost. Keywords used in the search strategy included "hypertension," "sleep duration," "Asian," and "adult." Boolean operators were used for the literature search. The search strategy is presented in Table 1.

**Table 1:** Search strategy

Database	Search Strategy
PubMed	("sleep duration"[MeSH Terms] OR ("sleep"[All Fields] AND "duration"[All Fields]) OR "sleep duration"[All Fields]) AND ("hypertense"[All Fields] OR "hypertension"[MeSH Terms] OR "hypertension"[All Fields] OR "hypertension s"[All Fields] OR "hypertensions"[All Fields] OR "hypertensive"[All Fields] OR "hypertensive s"[All Fields] OR "hypertensives"[All Fields]) AND ("asia"[MeSH Terms] OR "asia"[All Fields]) AND ("adult"[MeSH Terms] OR "adult"[All Fields] OR "adults"[All Fields] OR "adult s"[All Fields])
EBSCOhost	Sleep AND Asia AND Adult AND hypertension
Scopus	Sleep AND Asia AND Adult AND hypertension

Study Selection

Two independent reviewers (YASN and KW) conducted the initial screening of identified studies based on titles and abstracts. Subsequently, full-text reviews were performed to assess studies against the predetermined inclusion criteria. Any discrepancies were resolved through discussion, and if necessary, a third reviewer was consulted to reach a consensus.

Data Extraction

Two independent reviewers systematically extracted pertinent information from the included studies. The extracted data encompassed details, including the last name of the primary author, study year, geographical location, study type, baseline age, gender distribution, participant count, sleep assessment, hypertension ascertainment, categories of short sleep and normal sleep duration, and adjusted odd ratio (ORs) with corresponding 95% confidence intervals (CIs).

Risk of Bias Analysis

The risk of bias analysis for cohort studies was assessed using the Newcastle-Ottawa scale (NOS) [14,15]. Studies of cross-sectional design were assessed using Joanna Briggs Institute tools (JBI) [16].

Statistical Analysis

Our study employed random-effects models for quantitative synthesis, utilizing odds ratios (ORs) with corresponding 95% confidence intervals (CIs) to determine the overall association between short sleep duration and hypertension risk. Heterogeneity among studies was assessed using the I2 statistic, with values exceeding 50% indicating substantial heterogeneity.

Subgroup analyses, particularly focusing on gender-specific differences, were conducted to explore potential sources of heterogeneity and reveal variations in the association between short sleep duration and hypertension risk across different subgroups. All statistical analyses were executed using Review Manager 5.4. Statistical significance was established at a two-sided p-value less than 0.05.

Results

Study Selection

**Figure 1:**
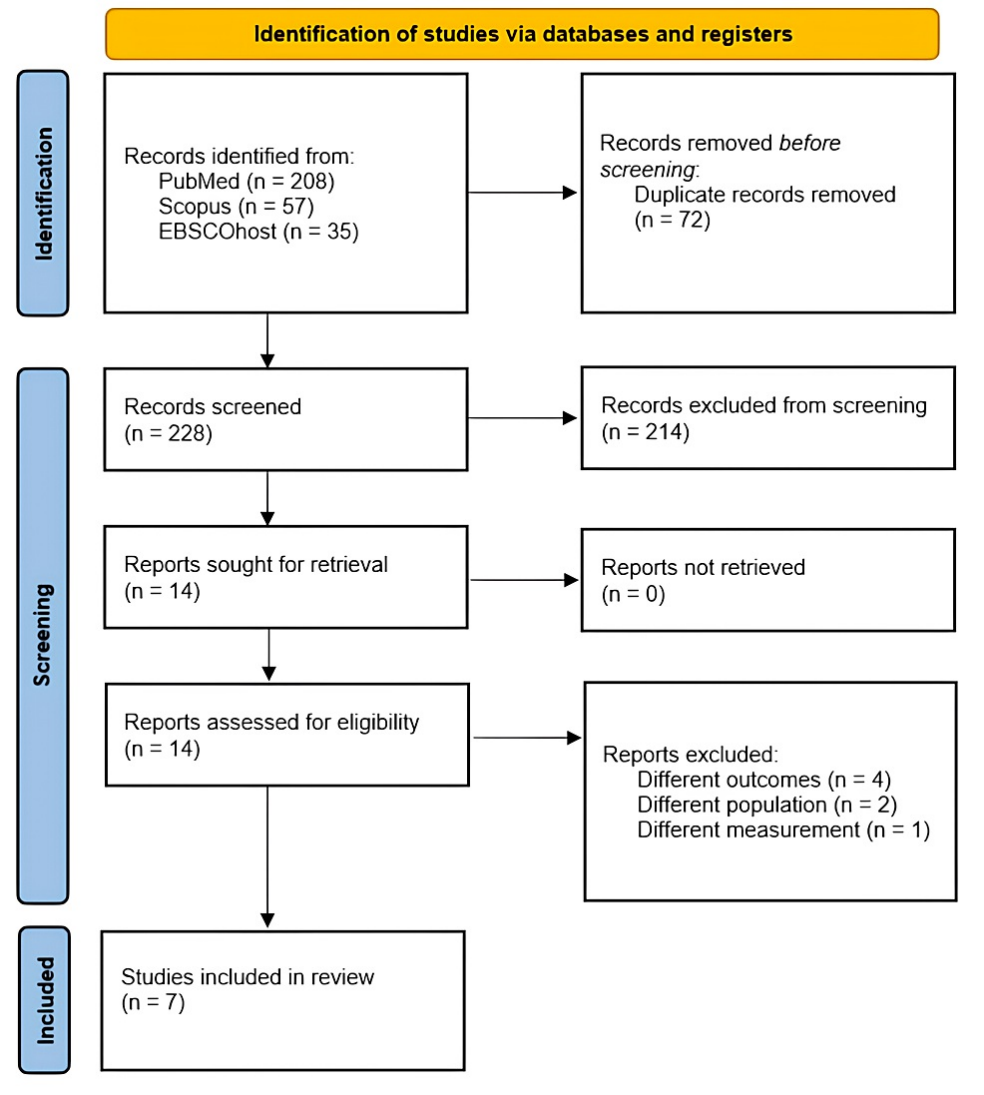
PRISMA flowchart for study selection PRISMA: Preferred Reporting Items for Systematic Reviews and Meta-Analyses

A flow diagram illustrating the study selection process, as per the Preferred Reporting Items for Systematic Reviews and Meta-Analyses (PRISMA) guidelines, is provided in Figure 1. Initially, a total of 228 articles were obtained after the removal of duplicates. Subsequently, 14 studies were selected for full-text assessment. Following the application of inclusion and exclusion criteria, seven studies were deemed eligible for inclusion in this review.

Study Characteristics

Table 2 summarizes the characteristics of the included studies. The included studies were published between 2010 and 2023. Geographically, the studies encompassed various Asian regions, including Korea [17,18], China [19-22], and Iran [23].

**Table 2:** Systematic review of sleep duration and the risk of hypertension

Author’s Name	Location	Study Type	Age (Years)	Female (%)	Participants	Sleep Assessment	Short Sleep Duration	Normal Sleep Duration
Kim et. al, 2010 [18].	Korea	Cross-sectional	19–99	57.8	5.393	Self-report	<5 h/day	7 h/day
Wang et. al, 2011 [21].	China	Cross-sectional	18–65	43.1	1.816	Self-report	<7 h/day	7-9 h/day
Yadav et. al, 2016 [17].	Korea	Prospective cohort	40-70	63.3	1.715	Questionnaire conducted with the help of trained interviewers	<6 h/day	6-7.9 h/day
Li et. al, 2019 [19].	China	Cross-sectional	18-79	53.0	19.407	Self-report	<7 h/day	7-8 h/day
Wu et. al, 2019 [22].	China	Cross-sectional	80-99	48.9	1.066	Self-report	<6 h/day	7-8 h/day
Yazdanpanah et. al, 2020 [23].	Iran	Retrospective cohort	35-70	Unclear	10.129	Pittsburgh Sleep Quality Questionnaire	<6 h/day	7 to 7.9 hours
He et. al, 2022 [20].	China	Cross-sectional	23-98	55.8	20.053	Self-report	<6 h/day	6-8 h/day

Methods employed for assessing sleep duration were all by self-report and a questionnaire. Hypertension ascertainment was conducted through office blood pressure measurement by healthcare providers. None of the studies used ambulatory blood pressure monitoring.

It should be noted that the studies use various definitions of short sleep duration. For example, the study by Kim et al. defined short sleep duration as <5 hours/day while other studies used the definition of < 6 hours/day to < 7 hours/day [13]. Furthermore, the definitions of normal sleep duration also vary between different studies.

Short Sleep Duration and Hypertension Risk

**Figure 2:**
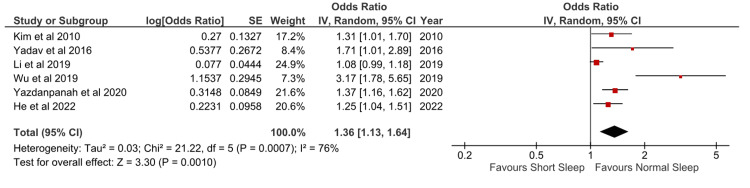
Meta-analysis comparing short versus normal sleep duration and hypertension risk Sources: [17-20,22,23]

A total of six studies were available for pooled analysis. The pooled analysis revealed a significant association between short sleep duration and hypertension risk among Asian adults, irrespective of sex. Figure 2 displays the forest plot depicting the pooled odds ratios (ORs) and corresponding 95% confidence intervals (CIs) across studies.

The overall pooled OR for hypertension risk associated with short sleep duration was 1.36 (95% CI:1.13-1.64). Substantial heterogeneity was observed among the included studies, with an I^2^ statistic of 76% (p: 0.0007). The high heterogeneity is likely to be caused by various definitions of short and normal sleep duration in the studies. Furthermore, some studies are of different designs (cross-sectional versus cohort).

The highest weight for the meta-analyses was from the study by Li et al. (2019) [19]. However, this study is the only study that did not find an association between short sleep duration and hypertension risk. Interestingly, this study defined short sleep duration as less than seven hours per day, which may suggest that seven hours may be the optimal cutoff for adequate sleep as other studies use the definition of short sleep duration below seven hours/day.

Subgroup Analysis

**Figure 3:**
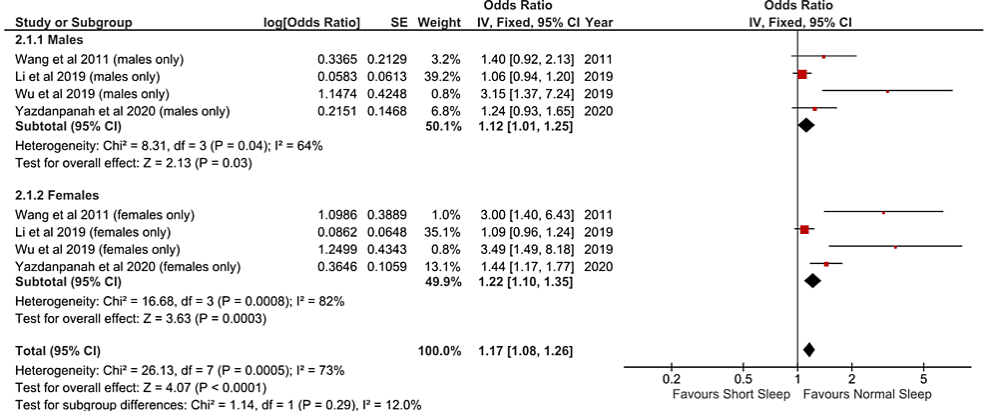
Subgroup analysis based on sex Sources: [17-23]

Subgroup analyses were conducted to explore potential sources of heterogeneity, focusing on the effect of sex. The Forest plot in Figure 3 illustrates the subgroup analysis results for males and females.

For males, the odds ratio (OR) was 1.12 (95% CI: 1.01-1.25), indicating a trend toward an increased risk of hypertension with short sleep duration. Similarly, among females, the odds ratio was 1.25 (95% CI: 1.10-1.35), indicating a significant association between short sleep duration and hypertension risk. However, the odds ratio in females is slightly higher than in males (1.25 versus 1.12).

The heterogeneity of pooled analysis for the male and female subgroups was 64% and 82%, respectively. The total combined pooled heterogeneity was 73%. As there is no significant change of heterogeneity after subgroup analysis based on sex, it is likely that sex is not a contributing factor to heterogeneity.

Risk of Bias

As the studies by Yadav et al. (2016) and Yazdanpanah et al. (2020) are cohort studies, they are appraised using the Newcastle-Ottawa Scale [17,23]. Based on the evaluation, the score given to the study by Yadav et al. and Yazdanpanah et al. is 8, which indicates a study with a low risk of bias [17,23].

Meanwhile, as other studies were cross-sectional studies, they were assessed using Joanna Briggs Institute (JBI) tool (Table 3). Based on the appraisal, only the study by He et al. (2022) analyzed confounding factors [20].

**Table 3:** Risk of bias analysis using the JBI tool for cross-sectional studies JBI: Joanna Briggs Institute

Questions	Studies				
	Kim et. al 2010 [7]	Wang et. al, 2011 [16].	Li et. al, 2019 [14].	Wu et. al, 2019 [17].	He et. al, 2022 [15].
Were the criteria for inclusion in the sample clearly defined?	Yes	Yes	Yes	Yes	Yes
Were the study subjects and the setting described in detail?	Yes	Yes	Yes	Yes	Yes
Was the exposure measured in a valid and reliable way?	Yes	Yes	Yes	Yes	Yes
Were objective, standard criteria used for measurement of the condition?	Yes	Yes	Yes	Yes	Yes
Were confounding factors identified?	No	No	No	No	Yes
Were strategies to deal with confounding factors stated?	No	No	No	No	Yes
Were the outcomes measured in a valid and reliable way?	Yes	Yes	Yes	Yes	Yes
Was appropriate statistical analysis used?	Yes	Yes	Yes	Yes	Yes

Discussion

This systematic review and meta-analysis sought to elucidate the relationship between sleep duration and hypertension risk within the adult Asian population. Our comprehensive analysis revealed a robust association between short sleep duration and increased hypertension risk, aligning with prior research highlighting the potential contribution of inadequate sleep to hypertension development. Notably, recent studies have underscored the significance of sleep duration in cardiovascular health, indicating that individuals with shorter sleep durations, particularly those sleeping less than six hours per night, face a notable 36% to 66% heightened risk of hypertension compared to those adhering to the recommended seven to eight hours of sleep [24]. Furthermore, emerging evidence suggests that both insufficient sleep (less than 7 hours) and excessive sleep (more than 8 hours) may elevate hypertension risk by 23% to 32%, as evidenced by a study involving a substantial cohort of 12,287 adults [25].

The mechanisms underlying the contribution of short sleep duration to hypertension are multifaceted and encompass various physiological processes. One prominent factor is physiological hyperarousal, particularly evident in individuals experiencing insomnia with heightened physiological arousal. This state of hyperarousal often coincides with increased sympathetic nervous system activity, fostering blood pressure elevation [26]. Furthermore, sleep deprivation disrupts the normal circadian rhythm of blood pressure regulation, accentuating daytime hypertension, particularly pronounced in individuals with compromised sleep quality [7,27]. Additionally, short sleep duration correlates with insulin resistance, fostering impaired glucose metabolism and elevating the risk of type 2 diabetes, thereby exacerbating hypertension development over time [6]. Genetic predispositions may also contribute, although the specific genes involved and their mechanisms remain elusive [28]. Experimental sleep restriction studies have revealed associations with increased calorie consumption, potentially contributing to obesity-related hypertension [7,29]. Endothelial dysfunction, characterized by attenuated endothelium-dependent nitric oxide production, is also implicated in the pathophysiology of hypertension, further underlining the intricate interplay between sleep duration and cardiovascular health [7,30]. Collectively, these factors underscore the complex interplay between sleep duration and hypertension, highlighting the need for comprehensive interventions targeting both sleep quality and duration to mitigate cardiovascular risk effectively.

Moreover, our study uncovered gender-specific patterns in the association between sleep duration and hypertension risk. Females exhibited a stronger association between short sleep duration and hypertension compared to males, a finding supported by previous research suggesting that females may lack baroreflex, a mechanism that detects increases in arterial pressure and reduces sympathetic nerve activity, which has a protective effect on blood pressure [8]. For instance, the Whitehall II study by Capuucio et al. revealed that shorter sleep duration was associated with a higher risk of hypertension in females, particularly those in the menopausal period who may experience frequent psychosocial problems and depression, contributing to shorter sleep duration [8]. Conversely, younger females, demonstrated an association between short sleep duration and hypertension, possibly due to sympathetic activation while older females did not exhibit this association due to age-related loss of arterial compliance [31]. These gender-specific differences underscore the importance of considering sex as a factor in understanding the relationship between sleep duration and hypertension risk.

Acknowledging the limitations of our study, we address several key concerns that may affect the validity and generalizability of our findings. Primarily, the reliance on self-reported sleep duration in the included studies introduces the possibility of recall bias and misclassification. Additionally, variations in the definitions of short and normal sleep durations across studies may influence the consistency of our results. Moreover, the high heterogeneity among studies underscores the need for a cautious interpretation of the pooled effect estimates. Lastly, the cross-sectional nature of most included studies limits our ability to establish causality between sleep duration and hypertension risk, highlighting the necessity for longitudinal investigations to elucidate temporal relationships.

Future research endeavors should aim to address these limitations and elucidate the underlying mechanisms linking sleep duration to hypertension risk in the Asian population. Longitudinal studies with objective measures of sleep duration, such as actigraphy or polysomnography, are warranted to establish temporality and causality. Furthermore, investigations into potential mediators and moderators of the sleep-hypertension relationship, including genetic, psychosocial, and environmental factors, are crucial for developing targeted interventions and personalized approaches for hypertension prevention and management in Asian adults.

## Conclusions

Based on the analyzed studies, short sleep duration is associated with a higher mild risk of hypertension, irrespective of sex. Thus, sleep duration can be a modifiable risk factor that can be prevented to reduce the risk of hypertension. By incorporating sleep hygiene practices and promoting healthy sleep habits, significant improvement in cardiovascular health can be made, especially in hypertension risk at a population level. Further studies on the effect of sleep duration in different age populations should be conducted to confirm the impact of short sleep duration.
